# Favorable safety profile of NOAC therapy in patients after tricuspid transcatheter edge-to-edge repair

**DOI:** 10.1007/s00392-024-02517-z

**Published:** 2024-08-19

**Authors:** Isabel A. Hoerbrand, Martin J. Kraus, Martin Gruber, Nicolas A. Geis, Philipp Schlegel, Norbert Frey, Mathias H. Konstandin

**Affiliations:** 1 Department of Internal Medicine III, Division of Cardiology, University Hospital Heidelberg, Ruprecht-Karls University Heidelberg, Im Neuenheimer Feld 410, 69120 Heidelberg, Germany; 2 DZHK (German Center for Cardiovascular Research), partner site Heidelberg/Mannheim, Heidelberg, Germany

**Keywords:** Transcatheter tricuspid valve repair, Transcatheter edge-to-edge repair, Tricuspid regurgitation, Oral anticoagulation, NOAC, Bleeding, Tricuspid disease

## Abstract

**Background:**

Transcatheter edge-to-edge repair for severe tricuspid regurgitation (TR) is a new treatment option (t-TEER). Data on optimal antithrombotic therapy after t-TEER in patients with an indication for anticoagulation are scarce and evidence-based guideline recommendations are lacking. We sought to investigate efficacy and safety of novel oral anticoagulation (NOAC) and vitamin-K-antagonists (VKA) in patients undergoing t-TEER.

**Methods:**

Among 78 consecutive patients with t-TEER of severe TR, 69 patients were identified with concomitant indication for oral anticoagulation. Outcomes of these patients treated with NOAC or VKA were compared over a median follow-up period of 327 (177–460) days.

**Results:**

Despite elevated thromboembolic and bleeding risk scores (CHA_2_DS_2_-VASc 4.2 ± 1.1, HEMORR_2_HAGES 3.0 ± 1.0 and HAS-BLED 2.1 ± 0.8), only one major bleeding incidence occurred under NOAC therapy. The risk for overall (NOAC 8% vs. VKA group 26%, *p = *0.044) and major bleeding events (NOAC 2% vs. VKA 21%, *p = *0.010) was significantly lower in the NOAC compared to the VKA group. No significant difference was found between NOAC and VKA treatment in terms of mortality (NOAC 18% vs. VKA 16%, *p = *0.865) or the combined endpoint of death, heart failure hospitalization, stroke, embolism, thrombosis, myocardial infarction, and severe bleeding (NOAC 48% vs. VKA 42%, *p = *0.801). A comparison between apixaban (*n = *27) and rivaroxaban (*n = *16) treated patients revealed no significant differences between NOAC substances (all bleeding events apixaban 7% vs. rivaroxaban 13%, *p = *0.638).

**Conclusion:**

Results of this study indicate that NOACs may offer a favorable risk–benefit profile for patients with concomitant indication for anticoagulation therapy following t-TEER.

**Graphical abstract:**

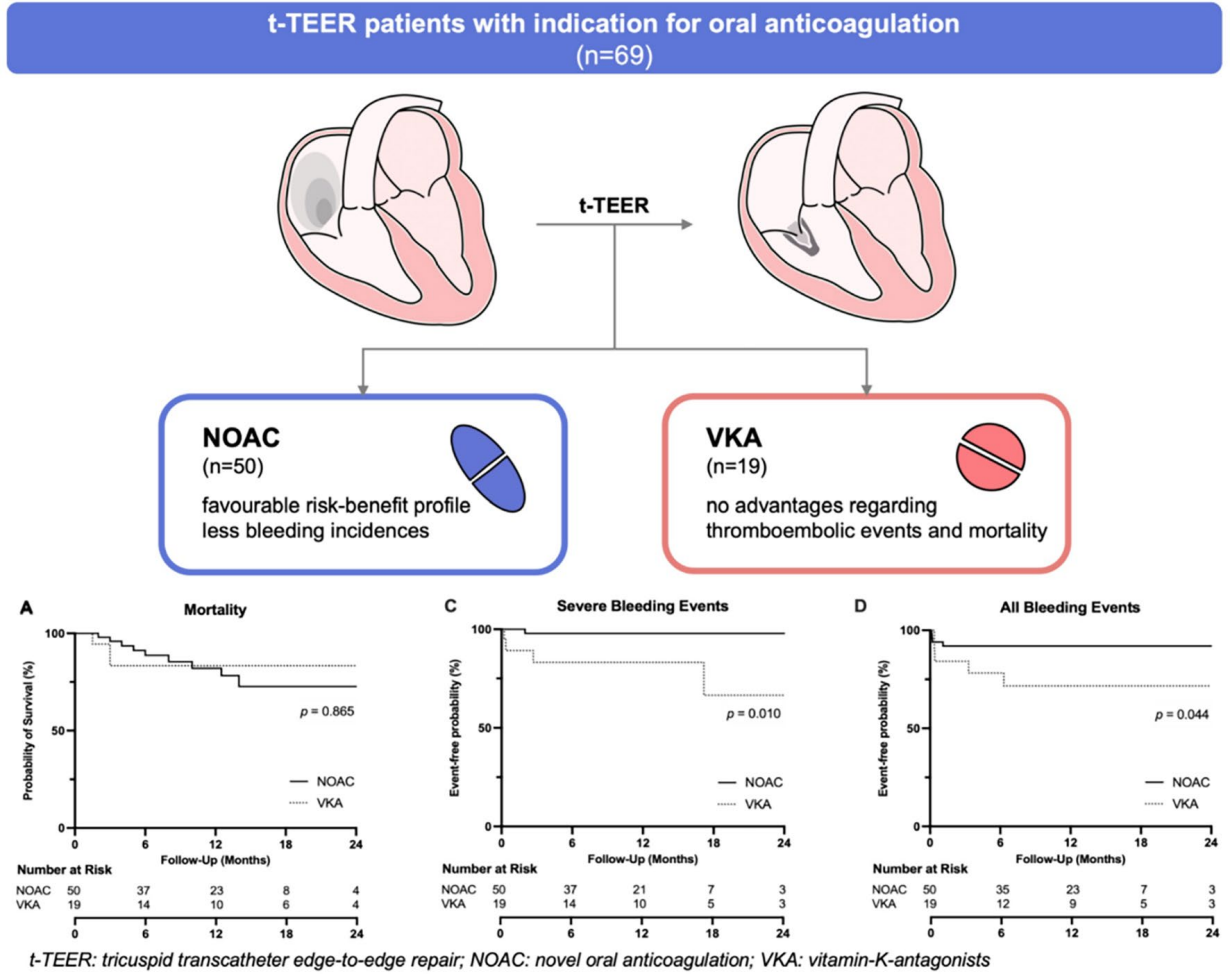

**Supplementary Information:**

The online version contains supplementary material available at 10.1007/s00392-024-02517-z.

## Introduction

Tricuspid regurgitation (TR) is a common valvular heart disease that often occurs secondarily in patients with chronic heart failure and is associated with a poor prognosis if left untreated [1, 2]. Transcatheter edge-to-edge repair (TEER) has emerged as a promising catheter-based treatment approach for higher-grade TR. Initial studies indicate a successful reduction in TR and clinical improvement following tricuspid TEER (t-TEER) [3, 4]. Severe TR is highly associated with atrial fibrillation, previous studies describe atrial fibrillation in over 80–90% of patients treated with TEER for TR [5, 6]. In clinical practice, usually preexisting oral anticoagulation therapy with NOAC or VKA is continued. However, data about optimal antithrombotic therapy after t-TEER are scarce and evidence-based guideline recommendations are lacking [7]. As these patients are often multimorbid, they are susceptible to both the risk of thromboembolic events and bleeding complications. Therefore, careful risk–benefit assessment is crucial to determine the most appropriate antithrombotic therapy.

NOACs have proven safety and efficacy in the prevention of thromboembolic complications in patients with non-valvular atrial fibrillation [8]. In recent years, several large prospective studies showed that NOACs have a favorable risk–benefit profile over VKA, with significant reductions in mortality and with similar risk of major bleeding [9–11]. In patients with mechanical valve replacement, VKAs remain the recommended anticoagulation strategy, due to higher rates of thromboembolic and bleeding events under NOACs [12]. The use of NOACs in patients undergoing t-TEER has not yet been evaluated. In this study, we provide first insight into efficacy and safety of NOAC therapy with concomitant indication for anticoagulation after t-TEER and compare outcomes with VKA therapy in the same patient population.

## Methods

### Patient characteristics

The study was performed as a retrospective observational analysis. From September 2020 to November 2022, 78 patients underwent successful TEER of severe or higher-grade TR. Sixty-nine patients were identified who had indication for anticoagulation therapy such as atrial fibrillation before t-TEER. Remaining nine patients had no indication for any OAC (see additionally Online Resource 1). Outcomes of 50 patients treated with NOAC were compared to 19 patients who received VKA over a median follow-up period of 327 days (interquartile range 177–460 days). Phenprocoumon was used as VKA. In a subgroup analysis, outcomes of patients treated with different NOAC substances were compared: apixaban was compared to rivaroxaban and other NOACs (apixaban *n = *27, rivaroxaban *n = *16, edoxaban *n = *5, Dabigatran *n = *2).

All patients presented with symptomatic, higher-grade TR and were considered not eligible for surgical repair due to high operative risk (see Table 1). Prior to intervention, periprocedural risk of all patients was estimated using EuroSCORE II. The decision on medical indication and technical feasibility for t-TEER was provided by our interdisciplinary heart team. Exclusion criteria in all patients comprised untreated severe aortic, mitral, or pulmonary valve disease, systolic pulmonary pressure >70 mmHg and expected survival <12 months.

**Table 1:** Baseline patient characteristics

Parameter	NOAC (*n = *50)	VKA (*n = *19)	*p*-value
Clinical data			
Age, years	80 (76; 83)	75 (63; 81)	**0.022**
Male sex, *n* (%)	24 (48)	11 (58)	0.592
BMI, kg/m^2^	26 ± 3	26 ± 5	0.704
NYHA FC	III (II–IV)	III (II–IV)	0.519
I, *n* (%)	–	–	
II, *n* (%)	9 (18)	2 (11)	
III, *n* (%)	37 (74)	15 (79)	
IV, *n* (%)	4 (8)	2 (11)	
Risk scores			
EuroScore II, %	4.2 (2.7; 6.2)	7.0 (4.3; 10.3)	**0.031**
HAS-BLED score	2.1 ± 0.8	2.1 ± 1.1	0.704
HEMORR_2_HAGES score	3.0 ± 1.0	3.2 ± 1.1	0.532
CHA_2_DS_2_-VASc score	4.2 ± 1.1	3.4 ± 1.3	**0.009**
Indication for anticoagulation			
Atrial fibrillation, *n* (%)	48 (96)	16 (84)	0.123
Mechanical valve	0	6 (32)	**<0.001**
Peripheral artery disease	0	1 (5)	0.275
Pulmonary embolism	1 (2)	0	>0.999
Peripheral thrombosis	1 (2)	0	>0.999
Comorbidity			
Arterial hypertension, *n* (%)	42 (84)	12 (63)	0.172
Significant CAD, *n* (%)	30 (60)	7 (37)	0.108
ICD/PM, *n* (%)	16 (32)	10 (53)	0.164
COPD, *n* (%)	7 (14)	6 (32)	0.164
Diabetes mellitus, *n* (%)	9 (18)	5 (26)	0.508
Renal insufficiency, *n* (%)	22 (44)	9 (47)	>0.999
Echocardiography			
Left ventricular ejection fraction, %	50 (45; 55)	43 (33; 54)	0.069
MAPSE, mm	13 ± 4	13 ± 3	0.578
TAPSE, mm	16 ± 4	14 ± 4	0.298
Right ventricle ED diameter, mm	47 ± 7	47 ± 5	0.996
Systolic pulmonary artery pressure, mmHg	45 (38; 55)	46 (45; 53)	0.223
TR grade	IV (III–IV)	IV (III–V)	0.832
I, *n* (%)	–	–	
II, *n* (%)	–	–	
III,* n* (%)	13 (26)	3 (16)	
IV,* n* (%)	26 (52)	13 (68)	
V,* n* (%)	11 (22)	3 (16)	

Values are given as absolute numbers and percent and mean ± SD or median and quartiles

*p* values are the results of a Mann–Whitney *U* test or *t* test according to standard distribution between NOAC and VKA group after t-TEER. *p* values of less than 0.05 were considered statistically significant and are presented in bold

*NOAC* novel oral anticoagulation, *VKA* vitamin K antagonist, *BMI* body mass index, *NYHA* New York Heart Association, *CAD* coronary artery disease, *ICD* implantable cardioverter defibrillator, *PM* pacemaker, *COPD* chronic obstructive pulmonary disease, *ED* end diastolic, *TR* tricuspid regurgitation, *TAPSE* tricuspid annular plane systolic excursion, *MAPSE* mitral annular plane systolic excursion

Primary endpoint was all-cause mortality. Secondary endpoints were severe bleeding complications, all bleeding complications, thromboembolic events (such as pulmonary embolism, ischemic stroke), heart failure hospitalization, device success, reduction of TR, and change in NYHA-classification. Bleeding complications and thromboembolic events were analyzed according to the Bleeding Academic Research Consortium (BARC)-classification [13].

### Procedure

Patients were treated under general anesthesia, and through transesophageal echocardiographic and fluoroscopic guidance. In this study, the PASCAL-Ace (*n = *65; Edwards, Irvine, CA, USA) or the TriClip (*n = *4; Abbott, Chicago, IN, USA) tricuspid valve repair systems were used for t-TEER. All interventional devices are CE-approved. Beginning, dosage and preparation of anticoagulation were administered according to standard operating procedure. During the procedure, an activated clotting time of 250–280 s was adjusted by heparin. In general, low-dose unfractionated heparin was administered intravenously the first post-procedural day. On day two after the procedure, VKA or NOAC therapy was initiated, VKA therapy was started under heparin bridging with PTT of 40–60 s until the target INR was reached (see Online Resource 2).

Procedural, technical, and device success were defined according to recommendations from the Mitral Valve Academic Research Consortium [14]. Procedural success denotes absence of major device or procedure-related serious adverse events; technical success, ability of the device to be deployed as intended and the delivery system successfully retrieved, and device success describes effectiveness of the device in reducing the severity of TR to optimal or acceptable levels.

A median of two devices were used per intervention (*p = *0.460). Deployment of the device was successful in 92% (NOAC) and 95% (VKA) of patients (technical success*, p* > 0.999). In one patient of the NOAC group, the second device could not be placed due to an inadequately wide gap. Procedural success was high in both groups (NOAC 100% vs. VKA 95%, *p* > 0.999). Device success could be achieved in most patients (NOAC 84% and VKA group 79%, *p* > 0.999) (see Online Resource 3).

### Echocardiographic workup

Patients were screened using transthoracic and 3D-transoesophageal echocardiography. Tricuspid regurgitation severity was evaluated using a recently proposed staging system, categorizing TR in five grades, ranging from “mild” to “torrential” [15]. Regurgitation area, vena contracta width, and variability as well as hepatic vein flow patterns were used to evaluate TR severity. Technical feasibility was assessed using transgastric and deep midesophageal views as previously described [16]. Transthoracic echocardiography at screening and follow-up was conducted according to current ASE/ESC guidelines [17].

### Statistical analysis

Continuous data are expressed as mean ± standard deviation for normal distributed or median and quartiles for non-normal distributed values. Categorical data are presented as absolute numbers and percentages. Baseline characteristics between groups were compared using Mann–Whitney *U* test, *t* test, Kruskal–Wallis test, or one-way ANOVA as required. Baseline and follow-up data were compared using Wilcoxon signed-rank test or paired Student’s *t* test as required. Log-rank test was used for survival time analyses. Graphics were designed using Prism GraphPad Software [18].

## Results

### Patient characteristics

Baseline patient characteristics of the 69 patients included in this analysis are presented in Table 1. Out of 69 t-TEER patients with concomitant indication for anticoagulation therapy, 50 patients were treated with NOACs and 19 patients received VKAs prior to the procedure. The anticoagulation regimen remained unchanged following t-TEER. All patients presented with highly symptomatic TR with a median NYHA functional class of III in both groups. Mean age was significantly higher in the NOAC compared to VKA group [NOAC 80 (76; 83) years vs. VKA group 75 (63; 81) years, *p = *0.022]. In both groups, the majority of patients were diagnosed with atrial fibrillation (NOAC 48/50 (96%) vs. VKA group 16/19 (84%), *p = *0.123). In the VKA group, seven patients had a contra indication against NOACs; six had undergone mechanic valve replacement and one patient received VKA due to severe peripheral arterial disease. Twelve patients received VKA therapy for atrial fibrillation. In these patients, VKA therapy was continued based on patient-specific factors, such as prior VKA stability, patient preference, or contraindication for NOAC therapy. Two patients received oral anticoagulation with NOACs for pulmonary embolism and peripheral thrombosis, respectively.

All patients had a significant bleeding and thromboembolic risk profile; HEMORR_2_HAGES [19] (NOAC 3.0 ± 1.0 vs. VKA group 3.2 ± 1.1, *p = *0.532) and HAS-BLED scores [20] (NOAC 2.1 ± 0.8 vs. VKA group 2.1 ± 1.1, *p = *0.704) were elevated, CHA_2_DS_2_-VASc score [21] was higher elevated in the NOAC group (NOAC 4.2 ± 1.1 vs. VKA group 3.4 ± 1.3, *p = *0.009). Mean EuroSCORE II was elevated in both groups. However, scores were higher in VKA patients [7.00 (4.30; 10.30)%] compared to patients treated with NOACs [4.20 (2.65; 6.23)%, *p = *0.031], estimating an intermediately elevated perioperative risk in all patients. Co-morbidities like arterial hypertension, significant coronary artery disease (CAD), device implantation, chronic obstructive pulmonary disease (COPD), diabetes mellitus, and renal insufficiency presented equally in both groups (Table 1).

Within echocardiographic work-up, left ventricular ejection fraction (LV-EF) was mildly reduced in both groups [NOAC 50 (45; 55)% vs. VKA group 43 (33; 54)%, *p = *0.069]. RV function was impaired (longitudinal right ventricular shortening: NOAC 16 ± 4 mm vs. VKA group 14 ± 4 mm, *p = *0.298) and end-diastolic RV diameter was enlarged (NOAC 47 ± 7 mm vs. VKA group 47 ± 5 mm, *p = *0.996) in all patients. Patients showed signs of pulmonary hypertension as systolic pulmonary artery pressure was elevated [NOAC 45 (38; 55) mmHg vs. VKA group 46 (45; 53) mmHg, *p = *0.223].

In the NOAC group, 27 patients were discharged with apixaban (21 patients 2 × 5 mg/day; six patients 2 × 2.5 mg/day), 16 patients were taking rivaroxaban (twelve patients 1 × 20 mg/day; four patients 1 × 15 mg/day), five edoxaban (four patients 1 × 60 mg/day; one patient 1 × 30 mg/day), and other two patients were treated with dabigatran (2 × 150 mg/day). In four NOAC patients, the dosage was adjusted to renal function during the index hospital stay. Baseline characteristics for analysis of different NOAC substances are shown in additional Online Resource 4. Patients treated with rivaroxaban had a significantly lower rate of chronic renal failure (rivaroxaban 19% vs. apixaban 52% and others 71%, *p = *0.027). There were no other differences in baseline characteristics.

### Follow-up

Patients were followed for a median of 327 days (interquartile range 177–460 days).

### Clinical outcome

The majority of all patients experienced clinical improvement in NYHA function class (NOAC 61% vs. VKA group 62%, *p = *0.971), without any difference between the NOAC and the VKA group (see Online Resource 5). Significant and consistent TR reduction to moderate or less could be achieved in a numerically but not significantly higher rate in NOAC patients (NOAC 86% vs. VKA group 71%, *p = *0.200).

### Mortality

In the NOAC group, nine patients (18%) and in the VKA group three patients (16%, *p = *0.865) died over the follow-up time. All-cause mortality rate did not significantly differ between both groups. None of the known causes of death were related to bleeding or thromboembolic events. In the NOAC group, reasons for death were therapy refractory heart failure (*n = *4), septicemia (*n = *2), cancer of unknown origin (*n = *1), and unknown reason (*n = *2). Reasons for death in the VKA group were heart failure (*n = *2) and unknown reason (*n = *1). One patient (5%) received implantation of left ventricular assistant device (LVAD) and was listed for heart transplantation. The rate of hospitalization due to cardiac decompensation was higher in the NOAC group, without reaching statistical significance (NOAC 48% vs. VKA 37%, *p = *0.369) (see Table 2).

**Table 2:** Outcome at follow-up

Events	NOAC	VKA	*p* value	Hazard ratio (95% CI)
Cardiovascular safety outcome	**(*n = *50)**	**(*n = *19)**		
Mortality	9 (18)	3 (16)	0.865	0.88 (0.24–3.18)
Heart failure hospitalization	24 (48)	7 (37)	0.369	0.70 (0.31–1.54)
All bleeding events	4 (8)	5 (26)	**0.044**	4.65 (1.04–20.77)
Major bleeding events (BARC-Grade ≥ 3)	1 (2)	4 (21)	**0.010**	13.00 (1.83–92.21)
Myocardial infarction	0	0	–	–
Stroke	0	0	–	–
Pulmonary or peripheral (thrombo-)embolism	0	0	–	–
Single leaflet detachment	3 (6)	1 (5)	0.906	–
NYHA FC improvement ≥ I	**(*n = *41)**	**(*n = *13)**		
	25 (61)	8 (62)	0.971	–
TR reduction to ≤ moderate	**(*n = *44)**	**(*n = *14)**		
	38 (86)	10 (71)	0.200	–

Values are given as absolute numbers and percent

*p* values result of comparison between NOAC and VKA therapy. Improvement in NYHA FC and TR reduction refer to all patients with complete three-month follow-up. *p* values of less than 0.05 were considered statistically significant and are presented in bold

*BARC* Bleeding Academic Research Consortium, *NYHA* New York Heart Association, *TR* tricuspid regurgitation

### Thromboembolic and bleeding events

Overall bleeding events occurred in 8% of patients in the NOAC group and 26% in the VKA group within the follow-up time after t-TEER (*p = *0.044). Minor bleedings associated to the intervention were mostly bleeding or aneurysm at the puncture site, which could be treated conservatively. One patient (2%) in the NOAC group and four (21%) in the VKA group experienced a major or life-threatening bleeding event (defined as BARC-Grade ≥ 3, *p = *0.010). One patient treated with NOAC experienced gastrointestinal hemorrhage with need of blood transfusion and vasoactive medication 58 days after tricuspid intervention. No major peri- or postinterventional bleeding complications occurred in the NOAC group. In the VKA group, two out of four hemorrhages (one venous bleeding with need for angiographic stenting intervention and one gastrointestinal bleeding) occurred peri-interventionally or within 72 h after t-TEER, respectively. Another patient suffered from significant macro-hematuria with need for intervention and transfusion 74 days post-interventional and another patient needed re-thoracotomy for hemothorax after LVAD implantation 510 days after t-TEER. According to these data, the incidence of all bleeding events (NOAC 4/50, 8% vs VKA group 5/19, 26%, *p = *0.044, HR 4.65) and severe bleeding events (NOAC 1/50, 2% vs. VKA group 4/19, 21%, *p = *0.010, HR 13.00) were significantly lower in the NOAC group.

There was no occurrence of thromboembolic events, such as stroke, pulmonary embolism, or peripheral thrombosis. Furthermore, no intra-cardiac thrombi or device thrombosis was detected in transthoracic and transesophageal echocardiograms.

### Combined endpoint

**Fig. 1:**
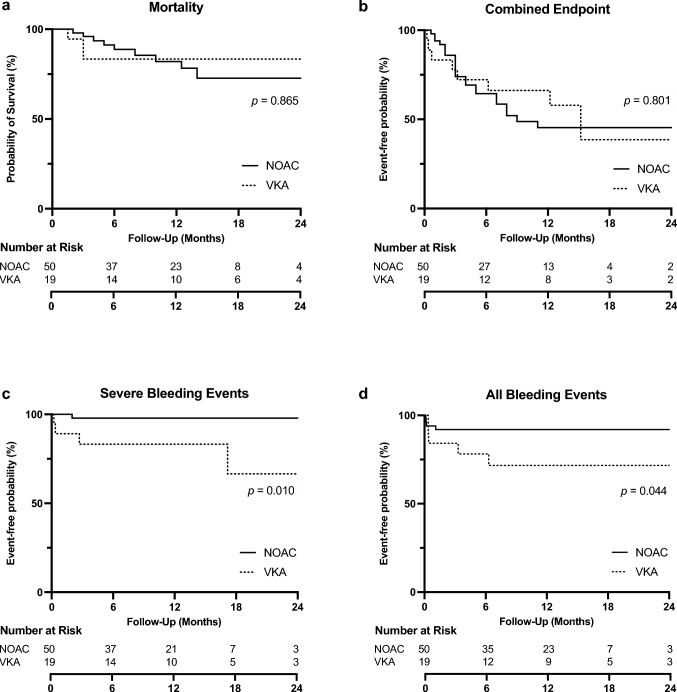
**a** Mortality, **b** combined endpoint, **c** severe and **d** all bleeding events in patients treated with NOAK or VKA after t-TEER: Combined end-point composes of death, heart failure hospitalization, stroke, (pulmonary) embolism, thrombosis, myocardiac infarction and severe bleeding. Severe bleeding events (life-threatening and major) were defined as BARC-Grade ≥ 3 (Bleeding Academic Research Consortium). *p* values indicate significance from log-rank test over a median follow-up period of 327 days (interquartile range 177–460 days). *NOAC* novel oral anticoagulation, *VKA* vitamin K antagonist, *t-TEER* tricuspid transcatheter edge-to-edge repair

Analysis of a combined endpoint of death, heart failure hospitalization, stroke, (pulmonary) embolism, thrombosis, myocardial infarction, and severe bleeding revealed a 48% (24/50) event rate in the NOAC group compared to a 42% (8/19) event rate in the VKA group. Thus, no statistically significant difference was observed (*p = *0.801) (Fig. 1).

### Comparison of different NOAC substances

Comparison of apixaban, rivaroxaban, and other NOAC substances revealed no significant difference in occurrence of severe (apixaban 0% vs. rivaroxaban 6% and other NOACs 0%, *p = *0.380) and overall bleeding events (apixaban 7% vs. rivaroxaban 13% and other NOACs 0%, *p = *0.638) (see Online Resource 6–8). One patient treated with rivaroxaban (1 × 15 mg/d) experienced a major bleeding event (gastrointestinal bleeding with need of blood transfusion and vasoactive medication) 58 days after t-TEER. Besides that, no major bleeding events and no thromboembolic events occurred.

Apixaban showed—compared to rivaroxaban and other NOACs—a tendency for higher rates of death (apixaban 22% vs. rivaroxaban 13% and others 14%, *p = *0.735) and combined endpoint of death, heart failure hospitalization, stroke, (pulmonary) embolism, thrombosis, myocardiac infarction, and severe bleeding (rivaroxaban 38% vs. apixaban 58% and others 29%, *p = *ns), without reaching statistical significance and notably in a small number of patients (*n = *16, 27 and 7, respectively).

## Discussion

This is the first study to retrospectively evaluate bleeding-associated and overall outcomes in patients with concomitant indication for oral anticoagulation after t-TEER for severe or higher-grade TR. Severe TR is, with up to 95%, highly associated with the presence of atrial fibrillation, as demonstrated by our and previous studies [4–6, 22]. While secondary TR is a multifactorial disorder, atrial fibrillation can lead to dilation of the right atrium, tricuspid annulus and base of the right ventricle, contributing to or causing significant TR. Anticoagulation therapy appears to be definitively appropriate when other co-morbidities with an indication for OAC are present. However, there is a lack of knowledge regarding the risks and benefits for anticoagulation therapy after t-TEER. The 2021 ESC/EACTS (European Society of Cardiology) and 2020 ACC/AHA (American College of Cardiology) guidelines did not provide a clear antithrombotic protocol for patients after mitral and tricuspid TEER [7, 23]. Consequently, anticoagulation policy has been based on clinical experience and was implicated individually in clinical practice.

A recent meta-analysis demonstrated that OAC therapy after mitral TEER (m-TEER) lowered the risk of stroke without an increase in the risk of bleeding, combined endpoints, or all‐cause mortality in patients using anticoagulants (at least 4-week duration), compared with those who did not receive anticoagulants [24]. The treatment regimen in the intervention group used mainly NOACs as anticoagulants, with or without the addition of varying antiplatelet agents. Optimal duration and choice of antithrombotic agents remain uncertain.

For t-TEER, there are no available data on antithrombotic therapy to this date [25]. For surgical bio-prosthetic valves, OAC therapy with VKAs is recommended for six months after tricuspid valve replacement [7, 23]. Of note, the risk of prosthetic valve thrombosis is greater for right-sided valves than left sided valves due to the relatively low-flow state in the right-sided circulation system.

In large studies, NOACs have proven a favorable risk–benefit profile for patients with non-valvular atrial fibrillation, leading to significant reductions in stroke, intracranial hemorrhage and mortality, however, with similar major bleeding and increased gastrointestinal bleeding risk compared to VKA [8]. The RE-ALIGN trial revealed elevated risks of stroke and major bleeding events in patients with mechanical heart valves when treated with the direct thrombin inhibitor dabigatran compared to VKA [12]. Consequently, NOACs are contraindicated in this patient cohort.

Patients included in this study were considerably sicker compared to those in the TRILUMINATE study [4]. For example, the percentage of patients with heart failure symptoms NYHA III or IV was 82% (NOAC) and 90% (VKA), respectively, compared to 59% in the TRILUMINATE study. Preceding percutaneous coronary artery intervention was at 60% (NOAC) and 37% (VKA) compared to 15% in the TRILUMINATE study. Kidney disease was present in 44% (NOAC) and 47% (VKA) compared to 35% of patients included in the TRILUMINATE study. Related to the more multi-morbid patient cohort in this study, we observed higher rates of mortality (NOAC group 18% and VKA group 16%) and hospitalization (NOAC 48% and VKA 36%) compared to the TRILUMINATE study. Most importantly however, in this study, only 2% of patients treated with NOACs, but 21% of patients treated with VKA experienced major bleeding events compared to 5,2% in the TRILUMINATE study within one year.

Overall bleeding rates following t-TEER were high in all patients—and especially under VKA therapy—(8% in the NOAC and 26% in the VKA group, *p = *0.024). These findings are consistent with the high bleeding risk noted in this particularly vulnerable and multi-morbid patient population (HEMORR_2_HAGES Score: NOAC 3.0 ± 1.0 vs. VKA group 3.2 ± 1.1, *p = *0.532 and HAS-BLED score: NOAC 2.1 ± 0.8 vs. VKA group 2.1 ± 1.1, *p = *0.704) and therefore emphasize the urgent need for optimal anticoagulation strategy.

In consistency with larger studies in the general population [8], we found favorable efficacy and safety profiles with NOAC therapy after t-TEER in comparison to VKA. NOAC therapy was associated with a significant lower risk of severe and overall bleeding events. Notably, severe bleeding events under NOAC therapy were observed only in one patient (2%). Although the NOAC group was older and had higher CHA_2_DS_2_-VASc scores, no evidence was found to suggest a higher risk of device thrombosis, pulmonary embolism, stroke or overall mortality compared to patients with VKA treatment. Out of the 50 patients treated with NOACs, nine (18%) died within median follow-up of 327 days, known causes of death were unrelated to bleeding or thromboembolic events. These findings suggest that NOAC therapy can be considered a safe and effective anticoagulation strategy for patients with a concurrent indication for anticoagulation therapy following t-TEER. In consistency with recent guidelines for anticoagulation in atrial fibrillation, NOACs should be prescribed preferably over VKA in these vulnerable patient cohort, providing there are no contraindication in this regard.

To evaluate possible differences between NOAC substances, we conducted a sub-analysis of patient outcomes treated with rivaroxaban in comparison to apixaban and other NOACs. We observed no significant differences concerning post-procedural severe and overall bleeding complications, thromboembolic occurrences, or all-cause mortality. Analysis of a composite end-point of post-procedural death, heart failure hospitalization, stroke, (pulmonary) embolism, thrombosis, myocardial infarction, and severe bleeding yielded similar results across all groups. In comparison to rivaroxaban and other NOACs, apixaban exhibited a propensity toward increased rates of mortality and combined endpoint although statistical significance was not attained. The predominantly prescription of apixaban for patients with chronic kidney disease suggests a potential correlation with the observed outcomes.

The topic of optimal anticoagulation management will further move into focus as new techniques for transcatheter tricuspid valve replacement (TTVR), such as the EVOQUE- and the TRICENTO system, are recently introduced [26, 27]. In ongoing studies, investigating these new TTVR approaches, VKA therapy is mostly administered in lack of data-based recommendations, resulting in high rates of severe and life-threatening bleeding events in up to 26% of patients one year after TTVR [28].

## Limitations

The present investigation is a retrospective study, conducted at a single center, and thus has some inherent limitations. The cohort size is limited, which may have impacted the adequate detection of differences in baseline parameters. Indication for anticoagulation was not uniform and due to the small cohort, there are no representative data on patients with initiation of OAC at the time of T-TEER. Patients in the VKA group had a higher EuroScore II and some had a contraindication for NOAC therapy such as mechanical valve replacement or severe peripheral artery disease. As a result, a higher target INR may have contributed to an increased risk of bleeding. However, this is the first study to examine the use of NOAC and VKA therapy after t-TEER in patients with a concomitant indication for OAC. Future large-scale, prospective and randomized studies are needed to validate our first observations.

## Conclusion

Severe and life-threatening bleeding incidences pose a serious risk in patients treated with t-TEER. Therefore, optimal anticoagulation therapy is crucial. Our findings provide initial data that NOACs have a favorable risk–benefit profile in patients with concurrent indication for anticoagulation therapy following t-TEER. We found both apixaban and rivaroxaban to have equal benefits although evidence from prospective, randomized studies in a greater number of patients is still lacking.

## Supplementary Information

Below is the link to the electronic supplementary material.

Supplementary file1 (DOCX 152 KB)
