# Supplementary material for: Favorable safety profile of NOAC therapy in patients after tricuspid transcatheter edge-to-edge repair
Source: Clin Res Cardiol. 2024 Aug 19;114(7):846–55. doi: 10.1007/s00392-024-02517-z (PMC12202579; doi:10.1007/s00392-024-02517-z)
Supplement: Supplementary file 1 — Supplementary file1 (DOCX 152 KB) [file 392_2024_2517_MOESM1_ESM.docx]

**Supplementary Material**

**Clinical Research in Cardiology**

**Favourable Safety Profile of NOAC Therapy in Patients after Tricuspid Transcatheter Edge-to-Edge Repair**

Isabel A. Hoerbrand, MD^1^; Martin J. Kraus, MD^1^; Martin Gruber^1^,

Nicolas A. Geis, MD^1^; Philipp Schlegel, MD^1^; Norbert Frey, MD^1,3^;

Mathias H. Konstandin, MD^1,3^

*^1^Department of Internal Medicine III, Division of Cardiology, University Hospital Heidelberg, Ruprecht-Karls University Heidelberg, 69120 Heidelberg, Germany*

*^3^DZHK (German Center for Cardiovascular Research), partner site Heidelberg/Mannheim, Germany*

Corresponding author: Dr. med. Mathias H. Konstandin

Department of Internal Medicine III, Division of Cardiology

Im Neuenheimer Feld 410, University of Heidelberg, 69120 Heidelberg, Germany

Phone:  +49 6221 56 35014; Fax:  +49 6221 56 5515

E-mail: [mathias.konstandin@med.uni-heidelberg.de](mailto:mathias.konstandin@med.uni-heidelberg.de)

Orchid: 0000-0001-9852-1039

**Supplementary Information 1** Patients without long-term OAC therapy

| **Patient** | **OAC Indication / Contraindikation** | **Medication** |
| --- | --- | --- |
| 1 | None, genetic risk factor for thrombophilia, factor V | aspirin |
| 2 | none | 4 weeks VKA, followed by aspirin |
| 3 | AF | aspirin, clopidogrel, individual decision |
| 4 | none | 4 weeks VKA, followed by aspirin |
| 5 | none, dialysis | 4 weeks heparin, followed by aspirin |
| 6 | none | 4 weeks VKA, followed by aspirin |
| 7 | none, frailty | aspirin |
| 8 | none | 4 weeks VKA, followed by aspirin |
| 9 | none | 4 weeks VKA, followed by aspirin |

**Supplementary Information 2** Anticoagulation before, peri- and procedural

|  | **Before Procedure** | **Periprocedural** | **24h postprocedural** | **48h post-procedural** | **Discharge** |
| --- | --- | --- | --- | --- | --- |
| **VKA** | Continuous heparin bridging | Heparin with activated clotting time of 250-280s | Continuous heparin bridging, partial thromboplastin time 40s | VKA under heparin bridging | VKA |
| **NOAC** | Pause 24h before procedure | Heparin with activated clotting time of 250-280s | Low-dose unfractionated heparin | NOAC | NOAC |

**Supplementary Information 3** Procedural Data. Values are given as absolute numbers and percent. *P*-values refer to NOAC and VKA group

| **Technical characteristics** | **NOAC**  **(n = 50)** | **VKA**  **(n = 19)** | ***P*-value** |
| --- | --- | --- | --- |
| **Number of implanted clips, n** 0.460 | | | |
| 1 (%) | 16 (32) | 9 (47) |  |
| 2 (%) | 29 (58) | 8 (42) |  |
| 3 (%) | 5 (10) | 2 (11) |  |
| **Implantation success** | | | |
| Procedural success | 50 (100) | 18 (95) | > 0.999 |
| Technical success | 46 (92) | 18 (95) | > 0.999 |
| Device success | 42 (84) | 15 (79) | > 0.999 |

**Supplementary Information 4** Baseline Patient Characteristics for NOAC Subanalysis. Values are given as absolute numbers and percent and mean ± SD or median and quartiles. *P*-values are the results of a Kruskal-Wallis test or one way ANOVA according to standard distribution between apixaban, rivaroxaban and other NOACs after t-TEER. *P*-values of less than 0.05 was considered statistically significant and are presented in bold. BMI, body mass index; NYHA, New York Heart Association; CAD, coronary artery disease; ICD, implantable cardioverter defibrillator; PM, pacemaker; COPD, chronic obstructive pulmonary disease; ED, end diastolic; TR, tricuspid regurgitation, TAPSE, tricuspid annular plane systolic excursion

| **Parameter** | **Apixaban**  **(n = 27)** | **Rivaroxaban**  **(n = 16)** | | **Other NOACs**  **(n = 7)** | ***P*-value** |
| --- | --- | --- | --- | --- | --- |
| **Clinical data** | | | | | |
| Age, years | 79 [74; 83] | 81 [78; 83] | | 82 [78; 87] | 0.354 |
| Male sex, n (%) | 13 (48) | 7 (44) | | 3 (57) | 0.946 |
| BMI, kg/m2 | 25 ± 3 | 26 ± 3 | | 27 ± 2 | 0.081 |
| **NYHA FC** | III (II-IV) | III (II-IV) | | III (II-III) | 0.906 |
| I, n (%) | - | - | | - |  |
| II, n (%) | 5 (19) | 3 (19) | | 1 (14) |  |
| III, n (%) | 20 (74) | 11 (69) | | 6 (86) |  |
| IV, n (%) | 2 (8) | 2 (13) | | - |  |
| **Risk scores** | | | | | |
| EuroScore II, % | 4.6 [2.6; 7.2] | 3.6 [2.2; 5.4] | | 4.9 [3.3; 9.6] | 0.399 |
| HAS-BLED score | 2.0 [2.0; 3.0] | 2.0 [2.0; 2.0] | | 2.0 [1.0; 3.0] | 0.621 |
| HEMORR₂HAGES score | 3.0 [2.5; 4.0] | 3.0 [2.0; 3.0] | | 3.0 [2.0; 4.0] | 0.357 |
| CHA₂DS₂-VASc score | 4.2 ± 1.14 | 4.2 ± 1.2 | | 4.3 ± 0.8 | 0.034 |
| **Comorbidity** | | | | | |
| Arterial hypertension, n (%) | 21 (78) | 15 (94) | | 6 (86) | 0.343 |
| Significant CAD, n (%) | 16 (59) | 8 (50) | | 6 (86) | 0.270 |
| Atral fibrillation, n (%) | 25 (93) | 16 (100) | | 7 (100) | 0.637 |
| ICD/PM, n (%) | 7 (26) | 6 (38) | | 3 (43) | 0.815 |
| COPD, n (%) | 5 (19) | 2 (13) | | 0 (0) | 0.422 |
| Diabetes mellitus, n (%) | 5 (19) | 3 (19) | | 1 (14) | 0.955 |
| Renal insufficiency, n (%) | 14 (52) | 3 (19) | | 5 (71) | **0.027** |
| **Echocardiography** | | | | | |
| Left ventricular ejection fraction, % | 50 [40; 55] | 50 [46; 55] | 54 [38; 55] | | 0.703 |
| TAPSE, mm | 17 ± 4 | 14 ± 5 | 14 ± 3 | | 0.088 |
| Right ventricle ED diameter, mm | 46 ± 6 | 49 ± 9 | 45 ± 4 | | 0.414 |
| Systolic pulmonary artery pressure, mmHg | 48 ± 12 | 44 ± 11 | 45 ± 12 | | 0.572 |
| **TR grade** | IV (III-IV) | IV (III-IV) | IV (III-V) | | 0.227 |
| I, n (%) | - |  | - | |  |
| II, n (%) | - |  | - | |  |
| III, n (%) | 4 (15) | 5 (31) | 3 (43) | |  |
| IV, n (%) | 16 (59) | 6 (38) | 4 (57) | |  |
| V, n (%) | 6 (22) | 5 (31) | - | |  |

**Supplementary Information 2** (A, B) TR grade and (C, D) NYHA functional class each in patients treated with NOAC and VKA pre and 3 months post t-TEER. NOAC, novel oral anticoagulation; VKA, vitamin K antagonist; NYHA, New York Heart Association; TR, tricuspid regurgitation

**Supplementary Information 6** (A) Mortality, (B) combined endpoint, (C) severe and (D) all bleeding events in patients treated with apixaban, rivaroxaban and other NOACs after t-TEER. Combined end-point composes of death, heart failure hospitalization, stroke, (pulmonary) embolism, thrombosis, myocardiac infarction and severe bleeding. Severe bleeding events (life threatening and major) were defined as BARC-Grade ≥ 3 (Bleeding Academic Research Consortium). P-values indicate significance from log-rank test. NOAC, novel oral anticoagulation; t-TEER, tricuspid transcatheter edge-to-edge repair

**Supplementary Information 7** NYHA FC grade improvement and TR reduction. Successful improvement in NYHA FC was defined as reduction in NYHA FC ≥ I post t-TEER. Successful TR reduction was defined as successful clip deployment and reduction to a TR grade of ≤ moderate at follow-up. Other NOACs unite Edoxaban and Dabigatran. NOAC, novel oral anticoagulation; VKA, vitamin K antagonist; NYHA, New York Heart Association; TR, tricuspid regurgitation

**Supplementary Information 8** Procedural Data and Outcome at Follow-up. Values are given as absolute numbers and percent. *P*-values are the results of a Kruskal-Wallis test or one way ANOVA according to standard distribution between apixaban, rivaroxaban and other NOACs after t-TEER. Improvement in NYHA FC and TR reduction refer to all patients with complete three months follow-up. BARC, Bleeding Academic Research Consortium. NYHA, New York Heart Association; TR, tricuspid regurgitation

| **Events** | **Apixaban**  **(n = 27)** | **Rivaroxaban**  **(n = 16)** | **Other NOACs**  **(n = 7)** | ***P*-value** |
| --- | --- | --- | --- | --- |
| **Implantation success** |  |  |  |  |
| Procedural success | 27 (100) | 16 (100) | 7 (100) | - |
| Technical success | 25 (93) | 14 (88) | 6 (86) | 0.456 |
| Device success | 23 (85) | 12 (75) | 7 (100) | 0.313 |
| **Cardiovascular Safety Outcome** | **(n = 27)** | **(n = 16)** | **(n = 7)** |  |
| Mortality | 6 (22) | 2 (13) | 1 (14) | 0.735 |
| Heart failure hospitalization | 15 (56) | 5 (31) | 2 (29) | 0.341 |
| All bleeding events | 2 (7) | 2 (13) | 0 | 0.638 |
| Major bleeding events  (BARC-Grade ≥ 3) | 0 | 1 (6) | 0 | 0.380 |
| Myocardial infarction | 0 | 0 | 0 | - |
| Stroke | 0 | 0 | 0 | - |
| Pulmonary or peripheral  (thrombo-) embolism | 0 | 0 | 0 | - |
| Single leaflet detachment | 1 (4) | 1 (6) | 1 (14) | 0.575 |
| **NYHA FC improvement ≥ I** | **(n = 21)** | **(n = 13)** | **(n = 7)** |  |
|  | 15 (71) | 7 (54) | 6 (86) | 0.312 |
| **TR reduction to ≤ moderate** | **(n = 27)** | **(n =16)** | **(n = 7)** |  |
|  | 19 (70) | 11 (69) | 6 (86) | 0.680 |
